# Tropical Australian Health-Data Linkage Shows Excess Mortality Following Severe Infectious Disease Is Present in the Short-Term and Long-Term after Hospital Discharge

**DOI:** 10.3390/healthcare9070901

**Published:** 2021-07-15

**Authors:** Oyelola A. Adegboye, Emma S. McBryde, Damon P. Eisen

**Affiliations:** 1 World Health Organization Collaborating Center for Vector-Borne and Neglected Tropical Diseases, College of Public Health, Medical and Veterinary Sciences, James Cook University, Townsville 4811, Australia; 2 Australian Institute of Tropical Health and Medicine, James Cook University, Townsville 4811, Australia; emma.mcbryde@jcu.edu.au (E.S.M.); damon.eisen@jcu.edu.au (D.P.E.); 3 College of Medicine and Dentistry, James Cook University, Townsville 4811, Australia

**Keywords:** sepsis, mortality, infectious disease, data-linkage, North Queensland

## Abstract

Background: In this study, we aimed to assess the risk factors associated with mortality due to an infectious disease over the short-, medium-, and long-term based on a data-linkage study for patients discharged from an infectious disease unit in North Queensland, Australia, between 2006 and 2011. Methods: Age-sex standardised mortality rates (SMR) for different subgroups were estimated, and the Kaplan-Meier method was used to estimate and compare the survival experience among different groups. Results: Overall, the mortality rate in the hospital cohort was higher than expected in comparison with the Queensland population (SMR: 15.3, 95%CI: 14.9–15.6). The long-term mortality risks were significantly higher for severe infectious diseases than non-infectious diseases for male sex, Indigenous, residential aged care and elderly individuals. Conclusion: In general, male sex, Indigenous status, age and comorbidity were associated with an increased hazard for all-cause deaths.

## 1. Introduction

Sepsis is a serious global health problem, with an estimated 11 million related deaths in 2017 [1]. In an attempt to emphasise the magnitude and complexity of the problem, in May 2017 the 70th World Health Assembly adopted a resolution [2]; ‘Improving the prevention, diagnosis and clinical management of sepsis.’ This resolution aims to bring more attention to sepsis, and describing patterns of severe infection and attendant mortality is an important component of this process.

In high-income countries, including Australia, communicable diseases mortality has reduced to the point where they are the main cause of only 3.0% of deaths [3]. However, data from the Australian Institute of Health and Welfare show that infectious and parasitic diseases frequently contribute to death as one of multiple causes, often in patients with chronic conditions. Infectious diseases of sufficient severity to require hospitalisation or occurring during hospitalisation increase both short- and long-term mortality relating to both the disease itself and other comorbidities [4]. A seven-year hospital-based prospective study performed in Australia reported additional infection-related mortality risk for patients who had sepsis managed in an intensive care unit [5].

Apart from information on incident causes of death, there are few long-term mortality studies in patient cohorts hospitalised due to infectious diseases. Incident causes of death data provide detailed information on community mortality, but additional insights accrue from the study of death among patient cohorts, including those who have been hospitalised. This information can help users to further target preventive measures like vaccination programs and the application of treatment guidelines.

We have used linked health data to examine mortality patterns of a cohort of patients discharged from a tropical Northern Australian hospital with a diagnosis of an infectious disease over an 11-year period [6]. We were particularly interested in assessing mortality that was directly due to severe infectious disease over the short-, medium-, and long-term to understand if preventive strategies may be of value to readdress any differences. As studies have shown that vulnerable groups have higher mortality [7,8,9,10,11,12], we conducted subgroup analysis to analyse any differences in mortality among Indigenous patients.

## 2. Materials and Methods

### 2.1. Data Sources

This cohort, subsequently referred to as TSV11, was comprised of 41,367 patients discharged over an 11-year period from the Townsville Hospital. Patients entered the cohort when they were discharged from the hospital with an International Classification of Diseases Version 10 Australian Modification (ICD-10-AM) code for infectious disease (Table S1) [6]. All TSV11 patient hospitalisation data prior to and after their index admission was available for analysis (between 2006 and 2016). The exit point was the final date of the study observation period for those alive at the end of the study (31 December 2016), and the date of death was extracted from the Queensland State Death Registry. For each death, date, causes of death, age, sex, comorbidities and Indigenous status were recorded as covariates. Causes of death (COD) as coded from the ICD-10-AM were grouped by the leading COD (Supplementary Tables S2 and S3). Patients were classified as Indigenous if they identified themselves as Aboriginal and/or Torres Strait Islander (ATSI) and non-Indigenous if they were neither Aboriginal nor TSI. We ranked patient illness severity based on the number and importance of comorbid diseases using the Charlson’s Comorbidity Index (CCI) (Supplementary Table S3) [13,14]. These data were available on cohort patients, so all were included in the analysis.

### 2.2. Data Analysis

The analyses in this study were three-fold. Firstly, we presented descriptive summaries for demographic and clinical characteristics as frequencies and percentages for categorical variables, and medians and interquartile range (IQR) for continuous variables. Chi-square test was used to compare the categorical variables between deaths due to severe infection and non-infective, and the Mann-Whitney U-test was used to test the differences in continuous variables. Secondly, we estimated the direct standardised age–sex specific death rates (ASR) separately for the ATSI and non-Indigenous populations using the procedure for computing direct standardised rates and risks ration “PROC STDRATE” in Statistics Analysis System software (SAS) version 9.4 [15,16] using the entire TSV11 patient cohort as the denominator. The population at risk and baseline death estimates by Indigenous status, age group and sex for Townsville for the calendar years 2006 to 2016 were obtained from the Australian Bureau of Statistics [17]. Yearly population data were aggregated and used as the total person-years over the 11-year period (2006–2016). We compared the ASR for the TSV11 cohort with that of the general Queensland population for the 11-year study period (2006–2016). In order to compare the incidence rates between groups and adjust for possible variation in age–sex distribution, standardised rate ratios (SRR) by age, sex and Indigenous status were calculated. Age groups were created as follows: ≤4, 5 to 14, 15 to 24, 25 to34, 35 to 44, 45 to 54, 55 to 64, 65 to 74 and 75+ years.

Thirdly, we focused on the primary outcome time from inclusion in the study to either date of death or final observation date in the study (31 December 2016) with censoring at status at last follow-up (Censored = 0, Dead = 1). The Kaplan-Meier method and log-rank test were used to estimate and compare the survival experience among different groups. We investigated the differences in the risk of deaths from severe infectious diseases, namely sepsis and pneumonia, in comparison to other non-infective diseases. Two senior infectious disease physicians (DPE and ESM) reviewed the ICD10 codes for cohort patients with infection and determined which should be included in this analysis as they represented severe infection. The following ‘severe infection disease’ ICD10-AM codes were included: bacteraemia, bacteria, septicaemia, fasciitis, infective, septic and pyelonephritis (Table S2).

Additionally, we investigated the risk factors for 30-day, 100-day and 11-year mortality based on the date of admission for the first infectious event. We analysed the competing risks of death due to severe infection and non-infective disease using cumulative incidence functions to calculate the proportion of patients suffering from each event over time [18]. The Fine and Gray method [19] was used for testing and estimating the hazard ratio (HR) for the sub-distribution (cause-specific) of competing risks for both severe infection death and non-infective death.

All statistical analyses were performed in SAS version 9.4 (SAS Institute, Cary, NC, USA) [16] and R version 3.6.2 (R Foundation for Statistical Computing) [20]. The 95% confidence intervals (CI) and two-tailed tests of significance are reported.

## 3. Results

### 3.1. Baseline Characteristics

The TSV11 database comprised a total of 41,367 patients during the period 1/1/2006 to 31/12/2016. Table 1 presents the descriptive summary of the 11-year, 100-day and 30-day mortality rates. During this 11-year period, 8274 (20% of the TSV11 cohort) died. Among these, 717 (8.7%) were residents of residential aged care facilities. The five most common primary COD were cancer (23%), cardiac disease (19.6%), severe infection (15.5%), sepsis (7.8%) and pneumonia (7.7%), and chronic pulmonary disease (8.6%). The overall median follow-up was 1356 days (IQR: 499–2409) and the median time to death was 380 days (IQR: 59–1165). The 30-day mortality rate was 1538 (3.7%) and with the highest proportions of deaths being due to infection (21.8%) compared with cancer (19.9%) and cardiac disease (18.3%) in this group. The overall 100-day mortality rate was 2590 (6.3%), with more deaths due to cancer (25.9%) than infectious (13.8%) or cardiac disease (17.2%). We further summarised the characteristics of the patients by severe infection COD or non-infective disease in Table S4. Patients who died of severe infections tended to have similar characteristics except for a shorter time to death in comparison with patients who died of non-infective diseases (*p*-value < 0.0001).

### 3.2. Standardised Mortality Rates

The all-cause crude mortality rate was 200 per 1000 persons, higher among non-Indigenous patients (210.7 per 1000 persons) than Indigenous patients (138.6 per 1000 persons) (Table S5). However, when the mortality rate was directly standardised for age and sex using the Townsville 2016 population as the reference, the age–sex standardised rate (ASR) among non-Indigenous patients (111.6 per 1000 persons) was lower than Indigenous patients (169.6 per 1000 persons) (Table 2), leading to an overall age-standardised risk ratio of for Indigenous patients of 1.5 (SRR = 1.5, 95%CI: 1.4–1.6). This was found across most age categories, the exceptions being the 5-14 and 75+ age groups (Table 2). When considering gender, the SRR mortality was higher in females than males across all ages except in the 5–14, 15–24, and 75+ age groups, where it was higher in males.

The ASR comparing all-cause deaths in the TSV11 cohort with that of the general Queensland population for the 11-year study period (2006–2016) is presented in Figure S1. All age group mortality rates were higher than those recorded in the general population. These ratios were highest among those aged 5 to 14 years and lowest in people aged 75+ years. Overall, the observed number of deaths (8274) was higher than the expected number of deaths (542.7), using Queensland as a reference population. This represents a standardised mortality ratio (SMR) of 15.3 (95%CI: 14.9, 15.6). The SMR broken down by age group and sex ranged from 5.7 (females aged 75+ years) to 60.2 (females aged 35–44 years) among Indigenous patients, and 7.7 (females aged 0–4 years) to 225.6 (females aged 5–14 years) among the non-Indigenous group (See Table 2).

### 3.3. Risk of Death Analysis

**Figure 1:**
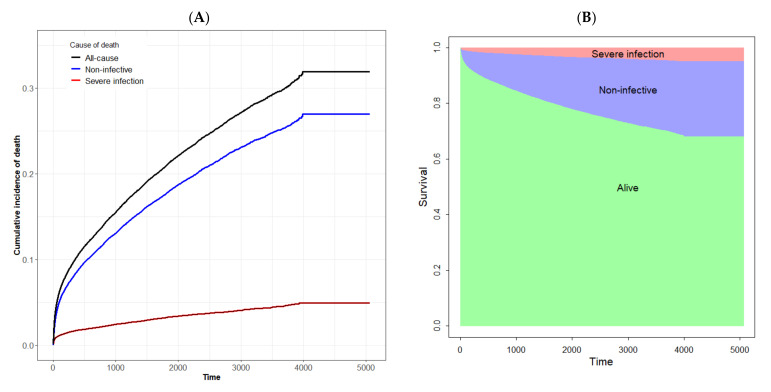
Distribution of patient’s survival over the 11-year study period from competing risk model. (**A**) Cumulative incidence rate, (**B**) Probability of survival.

Figure 1 presents the cumulative incidences of deaths from severe infection and non-infective diseases as well as the survival probability of the TSV11 cohort by cause of death. The sum of the cumulative incidences of severe infection and non-infective deaths equals the cumulative incidence of all-cause mortality (Figure 1A). The cumulative incidence of non-infective death exceeds that of severe infection throughout the study period. In Figure 1B, we present the Kaplan-Meier estimate of the survival function for the study cohort. The green area showed the probability of being alive, while the remaining areas were partitioned into death due to severe infection and non-infective disease. Overall, the probability of surviving to the end of the study period was 68.1% (95%CI: 67.0–69.2%), for 30 days was 96.3% (95%CI: 96.2–96.5%) and for 100 days was 93.7% (95%CI: 93.5–94.0%). The majority (5:1) of deaths were caused by non-infective diseases.

The 30-day, 100-day and overall (11-year) all-cause mortality risks adjusting for sex, age, residential age care, Indigenous status and degree of comorbidity based on the Charlson’s comorbidity index (CCI) are presented in Table 3. Considering all-cause mortality risks (30-day, 100-day and 11-year), male sex, identifying as Aboriginal and Torres Strait Islander, older age group and all measured comorbidity groupings increased the risk of death after adjusting for other variables. The mortality risk for being in residential aged care was protective for the 30-day (0.7, 95%CI: 0.6–0.9) and 100-day (0.7, 95%CI: 0.6–0.8) mortality, however, the risk increased by 20% (1.2, 95%CI: 1.1–1.3) for the 11-year study period. The risk of death associated with older age groups was more pronounced in long-term mortality (11-year) than short term (30 and 100-day) (Table 3).

We examined the estimated cause-specific hazard ratios for the risks of death associated with severe infection and non-infective diseases for 30 days and 100 days after hospitalisation and at the end of the 11-year study period. This revealed notable differences in the risk of death according to whether the cause was infective or non-infective. Table 4 presents the cause-specific hazard model for death due to severe infection and non-infective death for the 30-day, 100-day, and 11-year period. Being female reduced the cause-specific hazard of severe infection and non-infective deaths (HR: 0.85, 95%CI: 0.76–0.95) and (HR: 0.83, 95%CI: 0.80–0.88). On the other hand, cause-specific hazards for death due to severe infection continue to significantly decrease for females for the 11-year, 100-day, and 30-day mortalities but not for non-infective cause.

Living in a residential aged care facility increased the cause-specific hazard of severe infection death by 37% and non-infective death by 16% over the study period (Table 4). However, the risk associated with residential aged care facilities was protective for the 30-day and 100-day mortalities, especially for non-infective deaths. We found that residential aged care facility, older age, and being Indigenous had a more pronounced effect on the long-term cause-specific hazard of severe infection death than on non-infective death. Interestingly, the degree of comorbidities had more of an effect on non-infective deaths than severe infections, with severe comorbidities increasing the hazard of death more than fivefold in all time periods.

## 4. Discussion

We explored mortality patterns occurring in a cohort of patients discharged over an 11-year period (TSV11) from a tertiary hospital located in North Queensland with a diagnosis of an infectious disease. We found age-standardised mortality rates were higher for Indigenous than non-Indigenous Australians and, mostly, for males compared to females. We observed excess mortality in this hospital cohort compared to the general Queensland population, and, again, this was higher in Indigenous patients. The measured trend to increased mortality in patients managed in hospitals for infectious diseases has previously been noted in an Australian study [5]. Other studies have also reported a higher hospital mortality risk than the general population [5,21].

In this study, we assessed separately the risk factors for short-(30-day), mid-(100-day) and long-term (11-year) mortality for all-cause, severe infection and non-infective COD. Studies of long-term mortality exist for organ-specific infections such as community-acquired pneumonia [22] and sepsis syndrome [23]. These studies showed high rates of all-cause, respiratory infection and cardiovascular mortality. At least one study of long-term morbidity in community-acquired pneumonia used a population-based case-control methodology [22]. However, there are few studies of mortality for inpatient cohorts.

Previous studies have reported gaps in life expectancy between Indigenous and non-Indigenous people and a higher premature adult death among Indigenous peoples from Australia and New Zealand than non-Indigenous [8,9]. This has been attributed to historical and socio-economic factors [9,24]. By comparison, a Scottish study showed only a relatively small contribution of socio-economic disadvantage to increased mortality in large patient cohorts from Scottish hospitals [25]. We were able to confirm that mortality is higher among Indigenous patients in this cohort, however, we were not been able to advance the information about every specific cause for this observation. Among the TSV11 cohort of patients discharged from a Northern Australian hospital with an ICD-10-AM code for infectious disease, long-term mortality was more likely to be due to severe infection than non-infectious diseases for male sex, elderly, residential aged care and Indigenous people. This is consistent with previous observational cohort studies that found sepsis to be associated with an increased risk of deaths [26].

We have demonstrated a strong relationship between age groups and mortality. Survival rates declined significantly after the age of 14 years for short-, medium- and long-term mortality. As expected, patients older than 74 years had the highest hazard of death after adjusting for other variables. The differences in mortality risk for the older age group were notably higher for severe infection compared with non-infective COD, and more pronounced for long-term mortality. This is not surprising, as older age has been associated with longer hospital stays and infection disease due to the frailty and relatively impaired immune system function of these patients [27,28].

Multiple comorbidities measured by the increased Charlson comorbidity index (CCI) score were a strong significant predictor of deaths in this study. Although increasing CCI score is significantly associated with increased mortality risk, it was higher for non-infective deaths compared to severe infection and was also associated with long-term mortality. Previous studies found that increased comorbidity is a predictor of short- to long-term mortality [29,30].

In patients who reside in aged care facilities, we observed a reduction in the risk associated with short-term mortality for all-cause, severe infectious disease and non-infective COD [31]. However, expectedly, the risk of death increases for a longer observation period and is higher for severe infection than for non-infective COD. Research has indicated that frailty is a predictor of mortality and hospitalisation among residents of aged care facilities [31].

It is necessary to acknowledge the limitations of this study. While we used ICD10-AM codes to identify severe infectious and non-infectious disease, which has potential coding limitations, previous Australian research pertinent to our work showed very high agreement between ICD10-AM coding and chart reviews to confirm clinical diagnoses of pneumonia [32,33]. Many of the ICD codes that we used to define severe infection encoded sepsis. For example, A41 codes describe sepsis due to various pathogens. The presence of septic shock may be additionally coded as R57.2. As the data used were anonymised, we had no opportunity to review individual records to check whether the R57.2 septic shock code had been universally included by non-clinical hospital coders. With this in mind, we chose not to attempt to perform a formal sub-analysis on a possible link between septic shock and increased infectious diseases mortality. We would expect such an association to exist. This study was based on data sets from a single hospital in a regional area of the Australian tropics. We did not focus on tropical infectious diseases such as imported cases of malaria or dengue as the included ICD-10-AM discharge codes reflect severe infection due to all manner of infectious diseases. Overall, it must be kept in mind that any broader generalisation to other hospital populations, tropical or otherwise, must be made with caution.

Despite these limitations, the findings in this study emphasise the value of using a big health data approach, adding value through the reuse of hospital administrative and clinical databases. This approach has afforded an opportunity to calculate adjusted all-cause and severe infection mortality. This study contributed to the existing literature by exploring in more detail the mortality risk due to severe infections. The empirical analysis added to current research by showing that long-term mortality due to severe infection is higher for males, Indigenous people, those living in residential aged care and the elderly. It also showed that overall mortality is higher for the Indigenous population in a regional area than the general population.

## 5. Conclusions

This study is the first to describe long-term mortality in a cohort of patients admitted to a tropical Northern Australian hospital with severe infectious disease. We have shown that mortality in this cohort is substantially increased compared with the general population. Indigenous patients have a higher long-term mortality rate than non-indigenous individuals and for severe infection than non-infective deaths, respectively. This study further enumerates the health inequalities observed among subgroup communities and the need to ‘close the gap’. In conclusion, our findings suggest that long-term mortality risk is high for severe infection and among the Indigenous population. The health implications and burdens associated with sepsis and pneumonia, as demonstrated in this study, call for a community-based and well-coordinated strategy for the prevention and control of these infectious diseases in regional areas.

## Figures and Tables

**Table 1:** Demographic characteristics of the study cohort and COD, *n* (%) ^a^.

Characteristics	30-Day	100-Day	11-Year
Overall	1538 (3.7) ^a^	2590 (6.3) ^a^	8274 (20) ^b^
Follow-up, days ^c^	11 (5–20)	32 (11–64)	1356 (499–2409)
Time to death, days ^c^	10 (4–18)	22 (8–50)	380 (59–1165)
Gender			
Male	904 (58.8)	1508 (58.2)	4590 (55.5)
Female	634 (41.9)	1082 (41.8)	3684 (44.5)
Age group, years			
0–4	19 (1.4)	32 (1.2)	59 (0.7)
5–14	4 (0.3)	5 (0.2)	18 (0.2)
15–24	11 (0.7)	20 (0.8)	67 (0.8)
25–34	24 (1.6)	39 (1.5)	124 (1.5)
35–44	65 (4.2)	91 (3.5)	350 (4.2)
45–54	122 (7.9)	217 (8.4)	710 (8.6)
55–64	221 (14.4)	377 (14.6)	1237 (15.0)
65–74	354 (23.0)	600 (23.2)	1823 (22.0)
75+	718 (46.7)	1209 (46.7)	3886 (47.0)
Residential aged care	72 (4.7)	126 (4.9)	717 (8.7)
Indigenous status			
Non-Indigenous	1390 (90.4)	2372 (91.6)	7421 (89.7)
ATSI	148 (9.6)	218 (8.4)	853 (10.3)
Comorbidity Index			
Score 0	147 (9.6)	243 (9.4)	861 (10.4)
Score 1–2	432 (28.1)	715 (27.6)	2083 (25.1)
Score 3–4	429 (27.9)	664 (25.6)	1973 (23.9)
Score ≥ 5	530 (34.5)	968 (37)	3357 (40.6)
Cause of death (COD) ^d^			
*Severe Infection*			
Pneumonia	120 (7.8)	198 (3.1)	634 (7.7)
Sepsis	216 (14.0)	278 (10.7)	645 (7.8)
*Non-infective*			
Acute abdomen	30 (1.9)	37 (1.4)	113 (1.4)
Aspiration pneumonia	45 (2.9)	83 (3.2)	263 (3.2)
Cancer	306 (19.9)	670 (25.9)	1902 (23.0)
Cardiac	282 (18.3)	446 (17.2)	1621 (19.6)
Dementia	3 (0.2)	18 (0.7)	177 (2.1)
Haemorrhage	56 (3.6)	73 (2.8)	204 (2.5)
Liver	15 (1.0)	30 (1.2)	104 (1.3)
Multiple organ failure	61 (4.0)	79 (3.1)	198 (2.4)
Others	130 (8.5)	231 (8.9)	760 (9.2)
Pulmonary disease	133 (8.7)	217 (8.4)	708 (8.6)
Renal failure	50 (3.3)	91 (3.5)	386 (4.7)
Stroke	91 (14.0)	139 (10.7)	559 (6.8)

^a^ Percentage calculated from total deaths. ^b^ Out of the total patients. ^c^ Continuous variables summarised as median (Interquartile Range). ^d^ See Table S2.

**Table 2:** Overall mortality rates by Indigenous status during the study period of 2006–2011 compared with the Queensland population.

Gender	Age Group (Years)	Mortality Rates per 1000 Population						Standardised Mortality Ratio, TSV11 vs. Queensland					
		TSV11, 2006–2016			Queensland *, 2006–2016			ATSI			Non-Indigenous		
		ATSI	Non-Indigenous	SRR ^†^	ATSI	Non-Indigenous	SRR ^†^	SMR ^‡^	95%CI		SMR ^‡^	95%CI	
Female	0–4	25.3	5.9	4.3	1.2	0.8	1.5	20.8	9.9	31.7	7.7	2.9	12.5
	5–14	3.9	14.2	0.3	0.2	0.1	2.0	18.7	0	55.4	225.6	85.8	365.5
	15–24	10.8	9.3	1.2	0.6	0.3	2.0	19.1	3.8	34.4	38.1	21.0	55.3
	25–34	34.2	16.9	2.0	1.0	0.4	2.5	34.9	17.8	52	48.4	32.2	64.7
	35–44	157.4	56.4	2.8	2.6	0.7	3.7	60.2	46.2	74.3	78.9	62.2	95.6
	45–54	229.6	119.7	1.9	5.0	1.6	3.1	45.8	36.3	55.3	77.3	66.8	87.9
	55–64	367.6	204.3	1.8	12.5	3.6	3.5	29.4	23.9	34.8	56.9	50.9	62.9
	65–74	442.4	304.5	1.5	25.2	8.7	2.9	17.5	13.5	21.6	35.1	32.3	37.9
	75+	552.1	555.9	1.0	97.3	58.7	1.7	5.7	4.2	7.2	9.5	9.1	9.9
Male	0–4	19.7	10.8	1.8	1.1	1.1	1.0	18.2	8.7	27.7	10.3	5.9	14.7
	5–14	6.5	5.6	1.2	0.2	0.1	2.0	31.8	0	75.8	80.4	9.9	150.9
	15–24	37.9	18.6	2.0	0.9	0.5	1.8	44.1	19.1	69	38.9	25.0	52.8
	25–34	64.0	31.7	2.0	1.9	0.8	2.4	33.6	19.2	47.9	39.9	29.2	50.7
	35–44	171.4	72.0	2.4	4.3	1.5	2.9	40.1	30.9	49.3	48.2	39.6	56.8
	45–54	254.6	151.3	1.7	7.7	2.7	2.9	33.1	26.5	39.6	56.6	50.4	62.9
	55–64	384.6	268.9	1.4	17.3	5.9	2.9	22.3	17.7	26.9	45.4	42.1	48.8
	65–74	446.0	388.9	1.1	30.2	15	2.0	14.8	11.2	18.4	26.0	24.5	27.6
	75+	724.1	588.1	1.2	106.9	67.9	1.6	6.8	4.7	8.8	8.7	8.3	9.1
Overall		169.6	111.6	1.5	11.9	6.0	2.0	15.3					

Notes: ATSI = Aboriginal, Torres Strait Islander; Non-Indigenous = Non-Aboriginal, Torres Strait Islander; * Using 2016 death records. ^†^ Standardised Risk ratio (RRR): SRR greater than 1 implies mortality risk among ATSI is higher than non-Indigenous. ^‡^ SMR greater than one indicating that HC mortality rates are higher than expected compared to the Queensland group.

**Table 3:** Estimated overall survival all-cause hazards (HR) and 95%CI.

Characteristics	30-Day	100-Day	11-Year
Gender: Ref (Male)			
Female	0.8 (0.7–0.9)	0.8 (0.8–0.9)	0.8 (0.8–0.9)
Age: Ref (0–4 years)			
5–14	0.4 (0.2–1.2)	0.3 (0.1–0.9)	0.6 (0.4–1.1)
15–24	0.6 (0.3–1.2)	0.6 (0.4–1.1)	1.2 (0.8–1.6)
25–34	1.1 (0.6–1.9)	1.0 (0.6–1.6)	1.9 (1.4–2.6)
35–44	2.3 (1.3–3.9)	1.9 (1.3–2.9)	4.4 (3.3–5.8)
45–54	3.2 (2.0–5.3)	3.3 (2.2–4.8)	6.8 (5.2–8.9)
55–64	4.9 (3.0–7.9)	4.9 (3.3–7.1)	10.9 (8.4–14.3)
65–74	7.1 (4.4–11.5)	6.9 (4.8–10.0)	16.1 (12.3–21.0)
75+	11.5 (7.1–18.5)	11.3 (7.8–16.3)	31.7 (24.3–41.2)
Residential aged care	0.7 (0.6–0.9)	0.7 (0.6–0.8)	1.2 (1.1–1.3)
Indigenous: Ref (Non-Indigenous)			
ATSI	1.2 (1.0–1.4)	1.0 (0.8–1.2)	1.3 (1.2–1.4)
CCI: Ref (CCI score = 0)			
Mild: CCI score (1–2)	3.3 (2.7–4.1)	3.4 (2.9–3.9)	2.9 (2.7–3.1)
Moderate: CCI score (3–4)	4.3 (3.5–5.2)	4.0 (3.4–4.7)	3.6 (3.3–3.9)
Severe: CCI score (≥5)	4.2 (3.5–5.2)	4.8 (4.1–5.5)	4.9 (4.5–5.3)

Ref = Reference category; ATSI = Aboriginal and/or Torres Strait Islanders; CCI = Carlson Comorbidity index.

**Table 4:** Cause-specific hazard models for study cohort with 95%CI.

Characteristics	Severe Infection, HR (95%CI)			Non-Infective, HR (95%CI)		
	30-Day	100-Day	11-Year	30-Day	100-Day	11-Year
Gender: Ref (Male)						
Female	0.72 (0.57–0.90)	0.75 (0.62–0.90)	0.85 (0.76–0.95)	0.82 (0.73–0.92)	0.83 (0.76–0.91)	0.83 (0.80–0.88)
Age: Ref (0–4 years)						
5–14	0.36 (0.04–3.03)	0.37 (0.04–3.05)	0.53 (0.11–2.51)	0.48 (0.14–1.70)	0.32 (0.11–0.92)	0.66 (0.37–1.14)
15–24	0.18 (0.02–1.50)	0.18 (0.02–1.52)	0.79 (0.28–2.28)	0.76 (0.33–1.74)	0.71 (0.39–1.29)	1.20 (0.83–1.74)
25–34	1.14 (0.38–3.41)	1.71 (0.62–4.71)	2.47 (1.09–5.620)	1.05 (0.51–2.17)	0.85 (0.50–1.45)	1.81 (1.30–2.53)
35–44	1.47 (0.53–4.09)	2.07 (0.78–5.49)	4.32 (2.01–9.28)	2.65 (1.44–4.90)	1.84 (1.17–2.89)	4.33 (3.21–5.84)
45–54	2.56 (1.02–6.47)	4.00 (1.64–9.73)	7.30 (3.50–15.21)	3.46 (1.91–6.25)	3.05 (2.00–4.64)	6.66 (4.98–8.90)
55–64	3.69 (1.50–9.07)	6.20 (2.60–14.74)	13.03 (6.33–26.82)	5.25 (2.94–9.39)	4.46 (2.95–6.75)	10.54 (7.92–14.04)
65–74	6.05 (2.51–14.57)	9.73 (4.14–22.88)	22.57 (11.01–46.24)	7.51 (4.22–13.36)	6.24 (4.14–9.41)	15.06 (11.31–20.04)
75+	14.00 (5.91–33.13)	23.82 (10.26–55.29)	52.50 (25.72–107.17)	10.92 (6.16–19.38)	9.31 (6.19–14.00)	28.14 (21.17–37.41)
Residential aged care	0.68 (0.41–1.11)	0.91 (0.63–1.30)	1.37 (1.14–1.64)	0.71 (0.54–0.94)	0.65 (0.52–0.80)	1.16 (1.07–1.26)
Indigenous: Ref (Non-Indigenous)						
ATSI	1.21 (0.80–1.84)	1.32 (0.94–1.86)	1.44 (1.19–1.76)	1.20 (0.98–1.46)	0.96 (0.82–1.13)	1.24 (1.15–1.34)
CCI: Ref (CCI score = 0)						
Mild: CCI score (1–2)	1.86 (1.28–2.72)	1.62 (1.20–2.20)	2.13 (1.74–2.59)	4.06 (3.20–5.14)	4.18 (3.49–5.01)	3.06 (2.79–3.35)
Moderate: CCI score (3–4)	2.71 (1.86–3.93)	2.08 (1.53–2.83)	2.76 (2.26–3.36)	5.02 (3.94–6.39)	4.93 (4.10–5.92)	3.74 (3.41–4.11)
Severe: CCI score (≥5)	2.24 (1.54–3.26)	2.08 (1.54–2.80)	3.24 (2.67–3.92)	5.21 (4.11–6.61)	6.03 (5.04–7.21)	5.27 (4.825.76)

Ref = Reference category; ATSI = Aboriginal and/or Torres Strait Islanders; CCI = Carlson Comorbidity index.

## Data Availability

The research presented in this paper was supported by an Advance Queensland Research Fellowship.

## Supplementary Materials

The following are available online at https://www.mdpi.com/article/10.3390/healthcare9070901/s1, Figure S1. Mortality of Townsville Hospital database TSV11 cohort patients compared with age and sex-matched Queenslanders, Table S1. List of ICD-10-AM discharge codes for an infectious disease used to select the patient cohort, Table S2: Classification of major causes of deaths, Table S3. The ICD-10 codes used by comorbidity to compute the Charlson’s comorbidity index, Table S4. Characteristics of mortality by COD, Table S5. Summaries of the crude mortality rates by sex, age and Indigenous status.

## References

[1] Rudd K.E., Johnson S.C., Agesa K.M., Shackelford K.A., Tsoi D., Kievlan D.R., Colombara D.V., Ikuta K.S., Kissoon N., Finfer S. (2020). Global, regional, and national sepsis incidence and mortality, 1990–2017: Analysis for the Global Burden of Disease Study. Lancet.

[2] World Health Organization (2018). Improving the Prevention, Diagnosis and Clinical Management of Sepsis.

[3] Australian Institute of Health Welfare (2016). Australian Burden of Disease Study: Impact and Causes of Illness and Death in Australia 2011.

[4] Australian Institute of Health and Welfare (2020). Australian Institute of Health and Welfare 2019: Deaths in Australia.

[5] Ghelani D., Moran J.L., Sloggett A., Leeson R.J., Peake S.L. (2009). Long-term survival of intensive care and hospital patient cohorts compared with the general Australian population: A relative survival approach. J. Eval. Clin. Pract..

[6] Eisen D.P., McBryde E.S., Vasanthakumar L., Murray M., Harings M., Adegboye O. (2020). Linking administrative data sets of inpatient infectious diseases diagnoses in far North Queensland: A cohort profile. BMJ Open.

[7] Rowley K.G., O’Dea K., Anderson I., McDermott R., Saraswati K., Tilmouth R., Roberts I., Fitz J., Wang Z., Jenkins A. (2008). Lower than expected morbidity and mortality for an Australian Aboriginal population: 10-year follow-up in a decentralised community. Med. J. Aust..

[8] Hill K., Barker B., Vos T. (2007). Excess Indigenous mortality: Are Indigenous Australians more severely disadvantaged than other Indigenous populations?. Int. J. Epidemiol..

[9] Phillips B., Daniels J., Woodward A., Blakely T., Taylor R., Morrell S. (2017). Mortality trends in Australian Aboriginal peoples and New Zealand Māori. Popul. Health Metrics.

[10] Adegboye O.A., McBryde E.S., Eisen D.P. (2019). Epidemiological analysis of association between lagged meteorological variables and pneumonia in wet-dry tropical North Australia, 2006–2016. J. Expo. Sci. Environ. Epidemiol..

[11] Pak A., Eisen D., McBryde E., Adegboye O. (2021). Hospitalisation for lower respiratory tract infection is associated with an increased incidence of acute myocardial infarction and stroke in tropical Northern Australia. Sci. Rep..

[12] Pak A., Adegboye O.A., Eisen D.P., McBryde E.S. (2021). Hospitalisations related to lower respiratory tract infections in Northern Queensland. Aust. N. Z. J. Public Health.

[13] Charlson M.E., Pompei P., Ales K.L., MacKenzie C.R. (1987). A new method of classifying prognostic comorbidity in longitudinal studies: Development and validation. J. Chronic Dis..

[14] Quan H., Sundararajan V., Halfon P., Fong A., Burnand B., Luthi J.-C., Saunders L.D., Beck C.A., Feasby T.E., Ghali W.A. (2005). Coding algorithms for defining comorbidities in ICD-9-CM and ICD-10 administrative data. Med. Care.

[15] Yuan Y. (2013). Computing Direct and Indirect Standardized Rates and Risks with the STDRATE Procedure.

[16] SAS Institute Inc. (2017). SAS/STAT 14.3 User’s Guide.

[17] Australian Bureau of Statistics (2019). Deaths, Year of Occurrence, Age at Death, Age-Specific Death Rates, Sex, States, Territories and AUSTRALIA.

[18] Gooley T.A., Leisenring W., Crowley J., Storer B.E. (1999). Estimation of failure probabilities in the presence of competing risks: New representations of old estimators. Stat. Med..

[19] Fine J.P., Gray R.J. (1999). A proportional hazards model for the subdistribution of a competing risk. J. Am. Stat. Assoc..

[20] R Core Team (2017). R: A Language and Environment for Statistical Computing.

[21] Wright J., Plenderleith L., Ridley S. (2003). Long-term survival following intensive care: Subgroup analysis and comparison with the general population. Anaesthesia.

[22] Myint P.K., Hawkins K.R., Clark A.B., Luben R.N., Wareham N.J., Khaw K.T., Wilson A.M. (2016). Long-term mortality of hospitalized pneumonia in the EPIC-Norfolk cohort. Epidemiol. Infect..

[23] Davis J.S., He V., Anstey N.M., Condon J.R. (2014). Long term outcomes following hospital admission for sepsis using relative survival analysis: A prospective cohort study of 1,092 patients with 5 year follow up. PLoS ONE.

[24] Ring I.T., Firman D. (1998). Reducing indigenous mortality in Australia: Lessons from other countries. Med. J. Aust..

[25] Clark D., Armstrong M., Allan A., Graham F., Carnon A., Isles C. (2014). Imminence of death among hospital inpatients: Prevalent cohort study. Palliat. Med..

[26] Winters B.D., Eberlein M., Leung J., Needham D.M., Pronovost P.J., Sevransky J.E. (2010). Long-term mortality and quality of life in sepsis: A systematic review. Crit. Care Med..

[27] Khandelwal D., Goel A., Kumar U., Gulati V., Narang R., Dey A. (2012). Frailty is associated with longer hospital stay and increased mortality in hospitalized older patients. J. Nutr. Health Aging.

[28] Aw D., Woodrow L., Ogliari G., Harwood R. (2020). Association of frailty with mortality in older inpatients with Covid-19: A cohort study. Age Ageing.

[29] Rattanasompattikul M., Feroze U., Molnar M.Z., Dukkipati R., Kovesdy C.P., Nissenson A.R., Norris K.C., Kopple J.D., Kalantar-Zadeh K. (2012). Charlson comorbidity score is a strong predictor of mortality in hemodialysis patients. Int. Urol. Nephrol..

[30] Lu K., Kearney L.G., Ord M., Jones E., Burrell L.M., Srivastava P.M. (2013). Age adjusted Charlson Co-morbidity Index is an independent predictor of mortality over long-term follow-up in infective endocarditis. Int. J. Cardiol..

[31] Theou O., Sluggett J.K., Bell J.S., Lalic S., Cooper T., Robson L., Morley J.E., Rockwood K., Visvanathan R. (2018). Frailty, hospitalization, and mortality in residential aged care. J. Gerontol. Ser. A.

[32] Skull S.A., Andrews R.M., Byrnes G.B., Campbell D.A., Nolan T.M., Brown G.V., Kelly H.A. (2008). ICD-10 codes are a valid tool for identification of pneumonia in hospitalized patients aged > or = 65 years. Epidemiol. Infect..

[33] Skull S.A., Andrews R.M., Byrnes G.B., Campbell D.A., Kelly H.A., Brown G.V., Nolan T.M. (2009). Hospitalized community-acquired pneumonia in the elderly: An Australian case-cohort study. Epidemiol. Infect..

