# Supplementary material for: Tropical Australian Health-Data Linkage Shows Excess Mortality Following Severe Infectious Disease Is Present in the Short-Term and Long-Term after Hospital Discharge
Source: Healthcare (Basel). 2021 Jul 15;9(7):901. doi: 10.3390/healthcare9070901 (PMC8303504; doi:10.3390/healthcare9070901)
Supplement: Supplementary file 1 [file healthcare-09-00901-s001.zip › healthcare-1280869-supplementary.pdf]

Supplementary materials to “Excess mortality following severe infectious disease is present in long-term of hospital discharge: A Far North Queensland data-linkage study”

Oyelola A. Adegboye [1,2*], Emma S. McBryde [2] and Damon P. Eisen [2,3]

[1] World Health Organization Collaborating Center for Vector-Borne and Neglected Tropical Diseases, College of Public Health, Medical and Veterinary Sciences, James Cook University, Townsville 4811, Australia
[2] Australian Institute of Tropical Health and Medicine, James Cook University, Townsville 4811, Australia
[3] College of Medicine and Dentistry, James Cook University, Townsville 4811, Australia.
* Correspondence: oyelola.adegboye@jcu.edu.au; Tel.: +61 7 4781 5707

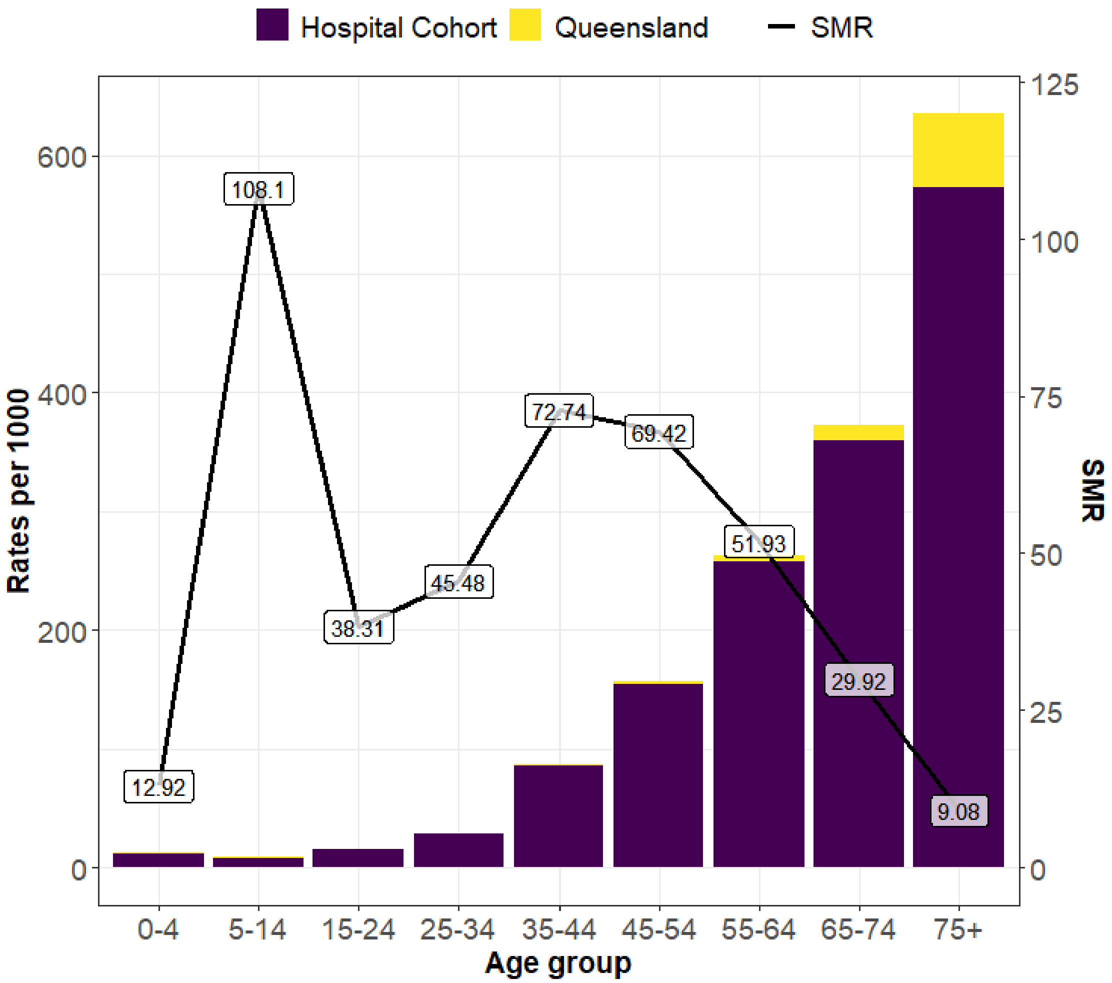

Figure S1. Mortality of Townsville Hospital database TSV11 cohort patients compared with age and sex-matched Queenslanders.

Table S1. List of ICD-10-AM discharge codes for an infectious disease used to select the patient cohort[1][†]

`	ICD10 codes
Infectious and parasitic diseases	A00–B99
Bacterial meningitis, not elsewhere classified	G00
Encephalitis, myelitis and encephalomyelitis	G04
Intracranial and intraspinal abscess and granuloma	G06
Intracranial and intraspinal phlebitis and thrombophlebitis	G08
Focal chorioretinal inflammation	H30.0
Purulent endophthalmitis	H44.0
Other endophthalmitis	H44.1
Disorders of vitreous body and globe	H45
Otitis Externa	H60
Mastoiditis	H70
Infective pericarditis	I30.1
Chronic constrictive pericarditis	I31.1
Pericarditis in diseases classified elsewhere	I30.0
Acute and subacute infective endocarditis	I33.0
Acute endocarditis unspecified	I33.9
Endocarditis and heart valve disorders in diseases classified elsewhere	I39
Infective myocarditis	I40.0
Myocarditis in diseases classified elsewhere	I41
Acute sinusitis	J01
Streptococcal pharyngitis	J02.0
Acute pharyngitis due to other specified organisms	J02.8
influenza and Pneumonia	J09-J18
Influenza due to certain identified influenza virus	J09
Influenza due to other identified influenza virus	J10
Influenza, virus not identified	J11
Viral pneumonia, not elsewhere classified	J12
Pneumonia due to Streptococcus pneumoniae	J13
Pneumonia due to Haemophilus influenzae	J14
Bacterial pneumonia, not elsewhere classified	J15
Pneumonia due to other infectious organisms, not elsewhere classified	J16
Pneumonia in diseases classified elsewhere	J17
Pneumonia, organism unspecified	J18
Peritonsillar abscess	J36
Retropharyngeal and parapharyngeal abscess	J39.0
Abscess of lung and mediastinum	J85
Pyothorax	J86.9
Acute peritonitis	K65.0
Disorders of peritoneum in infectious diseases classified elsewhere	K67
Abscess of liver	K75.0
Granulomatous hepatitis not elsewhere classified	K75.0
Liver disorders in infectious and parasitic diseases classified elsewhere	K77.0
Acute Cholecystitis	K81.0
Cellulitis	L03
Pyoderma	L08.0
Pyogenic arthritis	M00
Direct infections of joint in infectious and parasitic diseases classified elsewhere	M01
Post infective and reactive arthropathies in diseases classified elsewhere	M03
Necrotising Fasciitis	M72.6
Osteomyelitis	M86

UTI	N39.0
Inflammatory disease of the prostate	N41
Orchitis and epididymitis	N45.9
Fever of other and unknown origin	R50
Shock, not elsewhere classified	R57
Abnormal findings in cerebrospinal fluids	R83

†Reproduced with permission from BMJ Open (License Number 5106460775582)

Table S2: Classification of major causes of deaths

Cause of death	Description	ICD-10 codes
Severe infection		
Pneumonia/Sepsis	Chest infection, bronchopneumonia, lower respiratory tract infection, abscess, bacteraemia, bacteria, septicaemia, fasciitis, infective, influenza, septic, pyelonephritis	J09-J18/A40-A42
Non-infective		
Acute abdomen	Bowel obstruction, cholangitis, perforation, peritonitis, ischaemic bowel, ischaemic colitis	K56.6
Aspiration pneumonia	Aspiration pneumonitis	J69
Cancer	Cancer, carcinoma, metastatic, metastasis, myeloma, leukaemia, astrocytoma, lymphoma, glioblastoma, melanoma, tumor, mesothelioma	C00-C97
Cardiac	ischaemic heart disease, cardiomyopathy, arrythmia, cardiac failure, coronary, myocardial, asystole, ventricular failure<br>atherosclerosis, atrial fibrillation, cardiogenic shock,	I05-I09, I10-I15, I20-I25, I42, I43, I50, P29
Degenerative neurological disease	Alzheimer, parkinson, motor neurone disease	F00 - F03, F05.1, G20-G22, G30-G32
Haemorrhage	Bleed, haematemesis, varices, variceal, aneurysm, rupture, dissection, blood loss, exsanguination	K27.4, K92, I60, I62, P50
Liver	Hepatic, cirrhosis, alcoholic	B18, K70.0 - K70.3, K70.9, K71.3 - K71.5, K71.7, K73, K74, K76.0, K76.2 - K76.4, K76.8, K76.9, Z94.4, I85.0, I85.9, I86.4, I98.2, K70.4, K71.1, K72.1, K72.9, K76.5, K76.6, K76.7
Multiple organ failure	Multiple organ failure	R68.8
Pulmonary disease	Chronic lung disease, chronic pulmonary disease, airways disease, respiratory failure, respiratory failure emphysema, chronic bronchitis, interstitial lung,	I27.8, I27.9, J40 - J47, J60 - J67, J68.4, J70.1, J70.3
Renal failure	Kidney disease, tubular necrosis, uraemia	I12, I13.1, N03.2 - N03.7, N05.2 - N05.7, N18, N19, N25, Z49.0 - Z49.2, Z94, Z99.2
Stroke	Intracerebral haemorrhage, acute cerebrovascular accident (cerebrovascular accident stroke)<br>cerebral artery, brain infarct, cerebral haemorrhage, cerebral vascular, ischaemic attack, cerebrovascular, intracerebral, intracranial, transient	I64, I61, I63, I69

Table S3. The ICD-10 codes used by `comorbidity` to compute the Charlson's comorbidity index.

Description	ICD-10 codes	Charlson score
Myocardial infarction	I21.x, I22.x, I25.2	1
Peripheral vascular disease	I70.x, I71.x, I73.1, I73.8, I73.9, I77.1, I79.0, I79.2, K55.1, K55.8, K55.9, Z95.8, Z95.9	1
Congestive heart failure	I09.9, I11.0, I13.0, I13.2, I25.5, I42.0, I42.5 - I42.9, I43.x, I50.x, P29.0	1
Cerebrovascular disease	G45.x, G46.x, H34.0, I60.x - I69.x	1
Dementia	F00.x - F03.x, F05.1, G30.x, G31.1	1
Chronic pulmonary disease	I27.8, I27.9, J40.x - J47.x, J60.x - J67.x, J68.4, J70.1, J70.3	1
Rheumatic disease	M05.x, M06.x, M31.5, M32.x - M34.x, M35.1, M35.3, M36.0	1
Peptic ulcer disease	K25.x - K28.x	1
Mild liver disease	B18.x, K70.0 - K70.3, K70.9, K71.3 - K71.5, K71.7, K73.x, K74.x, K76.0, K76.2 - K76.4, K76.8, K76.9, Z94.4	1
Diabetes without chronic complication	E10.0, E10.1, E10.6, E10.8, E10.9, E11.0, E11.1, E11.6, E11.8, E11.9, E12.0, E12.1, E12.6, E12.8, E12.9, E13.0, E13.1, E13.6, E13.8, E13.9, E14.0, E14.1, E14.6, E14.8, E14.9	1
Diabetes with chronic complication	E10.2 - E10.5, E10.7, E11.2 - E11.5, E11.7, E12.2 - E12.5, E12.7, E13.2 - E13.5, E13.7, E14.2 - E14.5, E14.7	2
Hemiplegia or paraplegia	G04.1, G11.4, G80.1, G80.2, G81.x, G82.x, G83.0 - G83.4, G83.9	2
Renal disease	I12.0, I13.1, N03.2 - N03.7, N05.2 - N05.7, N18.x, N19.x, N25.0, Z49.0 - Z49.2, Z94.0, Z99.2	2
Any malignancy, including lymphoma and leukaemia, except malignant neoplasm of skin	C00.x - C26.x, C30.x - C34.x, C37.x - C41.x, C43.x, C45.x - C58.x, C60.x - C76.x, C81.x - C85.x, C88.x, C90.x - C97.x	2
Moderate or severe liver disease	I85.0, I85.9, I86.4, I98.2, K70.4, K71.1, K72.1, K72.9, K76.5, K76.6, K76.7	3
Metastatic solid tumour	C77.x - C80.x	6
AIDS/HIV	B20.x - B22.x, B24.x	6

Table S4. Characteristics of mortality by COD

Characteristics	Overall	Severe infection	Non-infective	P-value
Number	8274	1279 (15.5)	6995 (84.5)	
Days of follow-up: Median (IQR)	1356 (499 – 2409)	190 (13 – 967)	395 (65 – 1173)	<0.0001[a]
Days to death: Median (IQR)	380 (59 – 1165)	191 (14 – 968)	396 (66 – 1174)	<0.0001[a]
Age, continuous		74 (63 – 83)	73 (61 – 82)	0.1435[a]
Age group, years				
0-4	59 (0.7)	6 (0.9)	53 (0.7)	0.7987[b]
5-14	18 (0.2)	1 (0.2)	17 (0.2)	
15-24	67 (0.8)	3 (0.5)	64 (0.8)	
25-34	124 (1.5)	3 (0.5)	113 (1.5)	
35-44	350 (4.2)	11 (1.7)	326 (4.3)	
45-54	710 (8.6)	24 (3.7)	664 (8.7)	
55-64	1237 (15.0)	46 (7.13)	1143 (15.0)	
65-74	1823 (22.0)	145 (22.5)	1678 (22.0)	
75+	3886 (47.0)	315 (48.8)	3571 (46.8)	
Gender				
Male	4590 (55.5)	337 (62.3)	4253 (55.7)	0.0859[b]
Female	3684 (44.5)	308 (47.7)	3376 (44.3)	
Residential aged care	717 (8.7)	58 (9.0)	659 (8.6)	0.7588[b]
Indigenous status				
Non-Indigenous	7421 (89.7)	569 (88.2)	6852 (89.8)	0.2000[b]
ATSI	853 (10.3)	76 (11.8)	777 (10.2)	
Comorbidity Index				
Score 0	861 (10.4)	71 (11.0)	790 (10.4)	0.5192[b]
Score 1-2	2083 (25.1)	147 (22.8)	1936 (25.4)	
Score 3-4	1973 (23.9)	161 (25.0)	1812 (23.8)	
Score ≥ 5	3357 (40.6)	266 (41.2)	3091 (40.5)	

[a]Based on Mann-Whitney U-test

[b]Based on the Chi-square test

Table S5. Summaries of the crude mortality rates by sex, age and Indigenous status.

Gender	Age group (years)	11-year mortality, HC 2006-2016						Crude rates*
		Patients			No. of deaths			
		ATSI	Non-Indigenous	Total	ATSI	Non-Indigenous	Total	
Female	0-4	553	1690	2243	14	10	24	10.7
	5-14	255	705	960	1	10	11	11.5
	15-24	554	2036	2590	6	19	25	9.7
	25-34	468	2010	2478	16	34	50	20.2
	35-44	451	1524	1975	71	86	157	79.5
	45-54	392	1730	2122	90	207	297	140.0
	55-64	302	1694	1996	111	346	457	229.0
	65-74	165	1944	2109	73	592	665	315.3
	75+	96	3499	3595	53	1945	1998	555.8
Male	0-4	712	1940	2652	14	21	35	13.2
	5-14	310	895	1205	2	5	7	5.8
	15-24	317	1614	1931	12	30	42	21.8
	25-34	328	1670	1998	21	53	74	37.0
	35-44	426	1667	2093	73	120	193	92.2
	45-54	385	2082	2467	98	315	413	167.4
	55-64	234	2566	2800	90	690	780	278.6
	65-74	148	2808	2956	66	1092	1158	391.7
	75+	58	3139	3197	42	1846	1888	590.6
Overall		6154	35213	41367	853	7421	8274	200.0
Crude rates*					138.6	210.7		

Notes: ATSI= Aboriginal, Torres Strait Islander; Non-Indigenous = Non- Aboriginal, Torres Strait Islander; HC=Hospital cohorts. *Crude rates per 1000 persons

1. Eisen, D.P.; McBryde, E.S.; Vasanthakumar, L.; Murray, M.; Harings, M.; Adegboye, O. Linking administrative data sets of inpatient infectious diseases diagnoses in far North Queensland: a cohort profile. *BMJ Open* **2020**, *10*, e034845.
